# COVID-19 among immigrants in Norway, notified infections, related hospitalizations and associated mortality: A register-based study

**DOI:** 10.1177/1403494820984026

**Published:** 2021-01-07

**Authors:** Thor Indseth, Mari Grøsland, Trude Arnesen, Katrine Skyrud, Hilde Kløvstad, Veneti Lamprini, Kjetil Telle, Marte Kjøllesdal

**Affiliations:** 1 Norwegian Institute of Public Health, Health Services Research, Oslo, Norway; 2 Division of Infection Control and Environmental Health, Norwegian Institute of Public Health, Oslo, Norway

**Keywords:** COVID-19, immigrants, migrants, hospitalization, mortality

## Abstract

*Aim:* Research concerning COVID-19 among immigrants is limited. We present epidemiological data for all notified cases of COVID-19 among the 17 largest immigrant groups in Norway, and related hospitalizations and mortality. *Methods:* We used data on all notified COVID-19 cases in Norway up to 18 October 2020, and associated hospitalizations and mortality, from the emergency preparedness register (including Norwegian Surveillance System for Communicable Diseases) set up by The Norwegian Institute of Public Health to handle the pandemic. We report numbers and rates per 100,000 people for notified COVID-19 cases, and related hospitalizations and mortality in the 17 largest immigrant groups in Norway, crude and with age adjustment. *Results:* The notification, hospitalization and mortality rates per 100,000 were 251, 21 and five, respectively, for non-immigrants; 567, 62 and four among immigrants; 408, 27 and two, respectively, for immigrants from Europe, North-America and Oceania; and 773, 106 and six, respectively for immigrants from Africa, Asia and South America. The notification rate was highest among immigrants from Somalia (2057), Pakistan (1868) and Iraq (1616). Differences between immigrants and non-immigrants increased when adjusting for age, especially for mortality. Immigrants had a high number of hospitalizations relative to notified cases compared to non-immigrants. Although the overall COVID-19 notification rate was higher in Oslo than outside of Oslo, the notification rate among immigrants compared to non-immigrants was not higher in Oslo than outside. ***Conclusions:* We observed a higher COVID-19 notification rate in immigrants compared to non-immigrants and much higher hospitalization rate, with major differences between different immigrant groups. Somali-, Pakistani- and Iraqi-born immigrants had especially high rates.**

## Introduction

There is limited knowledge about groups at risk of COVID-19. Data from Sweden and Denmark suggest that immigrants from low- and middle-income countries have a higher risk of COVID-19 infection than non-immigrants [1–3]. In Denmark, these immigrants also have higher rates of COVID-19-related hospitalizations [3] and in Sweden, some immigrant groups, especially immigrants from Somalia, Syria and Iraq, have higher rates of COVID-19-associated mortality [1]. Studies from the UK and the US show that ethnic minorities tend to have higher incidence of COVID-19, and higher rates of COVID-19-related hospitalization and death than Whites [4–11]. Socioeconomic factors play an important role in the risk of COVID-19 and in differences between immigrant groups and host populations, but the excess risk among immigrants does not seem to be fully explained by differences in such factors or underlying health [8,9].

The first notified case of COVID-19 in Norway was reported on 26 February 2020. Throughout March, social distancing, hand and cough hygiene, travel advice and other preventive measures were introduced, and on 12 March the most radical measures since the Second World War, including school closure and travel bans, were imposed. In March the majority of identified cases were Norwegian-born tourists and their close contacts, most of them returning from skiing destinations in the Alps. Norway saw a decline in the number of new notified cases from end of March to the beginning of August. As numbers declined, the proportion of immigrants among new cases increased. Compared to most other countries in Northern Europe, Norway has had fewer notified cases, hospitalizations and deaths from COVID-19 [12].

Knowledge about groups at especially high risk is important to direct efforts to prevent disease transmission, severe illness and death and to plan and adapt health services accordingly. In Norway, we have a national surveillance of notified COVID-19 cases and of related hospitalizations and mortality, with country of birth registered for all cases. We utilize these data to describe the epidemiology of COVID-19 in Norway up to mid-July 2020. We aim to describe notified cases, related hospitalizations and associated mortality in the 17 largest immigrant groups in Norway, in immigrants from Europe, North America and Oceania (ENAO) and from Africa, Asia and South America (AfAsSA), and in non-immigrants, crude, age and sex adjusted. Together, this provides valuable insight about the proportion of notified cases and severity of infection in different groups.

## Methods

### Data sources, epidemiological surveillance

Since 31 January 2020, physicians and laboratories have been obligated to report all confirmed cases of COVID-19 with epidemiological, clinical and microbiological information to the Norwegian Surveillance System for Communicable Diseases (MSIS) using standard case-based notification practices [13–15]. As part of the legally mandated responsibilities of The Norwegian Institute of Public Health (NIPH) during epidemics, a new emergency-preparedness register covering the entire Norwegian population, was established in April 2020 [16]. In cooperation with the Norwegian Directorate of Health, individual-level data from several electronic administrative sources from 1 January 2020 (MSIS and Norwegian Patient register) were compiled and linked at the individual level using the unique personal identification number provided to everyone in Norway at birth or on immigration. The purpose of the preparedness register is to provide a rapid overview and knowledge of how the pandemic and the measures that are implemented to contain the spread of the virus affect the population’s health, use of healthcare services and health-related behaviours. The register contains daily updated information from the administrative record systems of all hospitals in Norway and daily updates from MSIS. COVID-19-related mortality in hospital is recorded on electronic systems and death – regardless of where it occurs – is notifiable to the MSIS. Aggregate data on the number of immigrants, by country of birth and age groups, were collected online at Statistics Norway (ssb.no).

### Variables

An *immigrant* is defined here as a person born outside Norway, but residing in Norway with legal residence. By defining immigrants by county of birth, persons born abroad by Norwegian parents fall into this category, but the number of such cases are too small to have any influence on estimates. A *non-immigrant* is a person born in Norway with permanent residence. Country of birth for non-residents cannot be identified in the data (and is excluded).

We focus on non-immigrants and immigrants from the 17 countries with more than 15,000 persons living in Norway at the beginning of 2020 (Poland, Sweden, Lithuania, Syria, Germany, Somalia, The Philippines, Denmark, Thailand, Iraq, Eritrea, Pakistan, the UK, Iran, Russia, Afghanistan and Romania). In addition, we report numbers for immigrants in total, and have categorized immigrants by region of origin, ENAO and AfAsSA. Missing information on country of birth is set to Norway. Age was recorded in groups (0–19, 20–69, ⩾70).

*COVID-19-associated mortality* is defined as when a person has died within 30 days after testing positive for COVID-19. *COVID-19-related hospitalization* is defined (according to national standards) as when a person has tested positive for COVID-19 and been hospitalized (inpatient) at a hospital in Norway during the 2 days before the 14-day period after the positive test.

### Study population

Our study population includes every person residing in Norway on 1 March who has a personal identification number in the Norwegian population register. Our study does not include workers, tourists and others on short-term stays or migrants without legal residence. Positive tests for SARS-CoV-2 were included up to 18 October 2020. To capture hospitalization and mortality according to our definitions, the study population was followed for 30 days after this date.

### Data analysis

Data on notified COVID-19 cases and associated hospitalizations and mortality were obtained from the preparedness register according to the definitions described above. Incidence rates were calculated as events*(100,000/population). Immigrant groups have an uneven age distribution compared to non-immigrants and risk of dying from COVID-19 is higher among the elderly [17]. Thus, we reported results that were both crude and age adjusted, using the direct standardization method, with non-immigrants the reference group. Due to relatively small numbers of notified cases and hospitalizations in each immigrant group, we chose to analyse women and men together, but we present numbers of notified cases, hospitalizations and mortality that are both crude and adjusted for age and sex. Oslo has had the highest notification rate of COVID-19 in Norway and it is the city with the highest proportion of residents who are immigrants. To elucidate whether differences in notified cases between immigrants and non-immigrants could be due to these attributes of Oslo, we compared the rates of confirmed cases among immigrants and non-immigrants in Oslo to respective rates outside Oslo.

Data handling and analyses were performed in Stata version 16.1 (StataCorp) and R version 3.6.2. Institutional board review was conducted and the Ethics Committee of South-East Norway confirmed (4 June 2020, #153204) that external ethical board review was not required.

## Results

Up to 18 October, 11,301 cases of COVID-19 were notified among non-immigrants, 4931 among immigrants in total, 1999 among immigrants from the ENAO region and 2932 among immigrants from the AfAsSA (Table I) region. From those, 964 hospitalizations and 222 deaths were reported among non-immigrants, 535 hospitalizations and 31 deaths among immigrants in total, 132 hospitalizations and nine deaths among immigrants from the ENAO region and 403 hospitalizations and 22 deaths among immigrants from the AfAsSA region (Table I). Compared to their share of the population, immigrants from the AfAsSA region had almost a three times higher rate of notified cases (773 versus 251 per 100,000) (Table I). Notified cases per 100,000 people were eight times higher for immigrants from Somalia (2057), seven times higher among immigrants from Pakistan (1868) and much higher among immigrants from Iraq (1616), Afghanistan (1391) and Iran (890) than for non-immigrants (251). Also, immigrants from Russia, Romania, Poland, the Philippines, Eritrea and Sweden had high rates of confirmed cases. Immigrants from Lithuania (163), Thailand (185), Germany (212) and Syria (243) had fewer cases per 100,000 than non-immigrants (Table I).

**Table I.** Notified COVID-19 cases and related hospitalizations (actual numbers and per 100,000) and median age at COVID-19-related hospitalization, according to immigrant background.

	*N*	Mean age (years) in group	Notified cases (*N*)	Notified cases per 100,000	Hospitalizations (*N*)	Hospitalizations per 100,000	Median age (year) at hospitalization	Mortality (*N*)	Mortality per 100,000
Non-immigrants	450,801	41	11,301	251	964	21	66	222	5
Immigrants	869,442	39	4931	567	535	62	53	31	4
ENAO region	489,921	41	1999	408	132	27	55	9	2
AfAsSA region	379,521	37	2932	773	403	106	52	22	6
Poland	101,736	39	498	490	7	7	53		
Sweden	47,180	44	202	428	11	23	58		
Lithuania	41,079	36	67	163	5	12	38		
Syria	32,450	28	79	243	15	46	42		
Germany	28,364	44	60	212	–		–		
Somalia	28,364	35	570	2057	98	354	50		
Philippines	25,083	37	119	474	18	72	50		
Denmark	24,223	49	81	334	12	50	76		
Thailand	22,242	37	43	185	7	30	47		
Iraq	22,706	40	367	1616	35	154	50		
Eritrea	22,077	32	103	467	11	50	43		
Pakistan	21,628	45	404	1868	68	314	63		
UK	20,624	45	55	267	–		–		
Iran	18,657	43	166	890	20	107	56		
Russia	18,333	41	128	698	17	93	52		
Afghanistan	16,968	32	236	1391	14	83	52		
Romania	15,599	37	83	532	–		–		

ENAO: Europe, North America and Oceania; AfAsSA: Africa, Asia and South America.

Numbers under five are not reported. Mortality (*N*) for each immigrant group is not reported.

**Figure 1.**
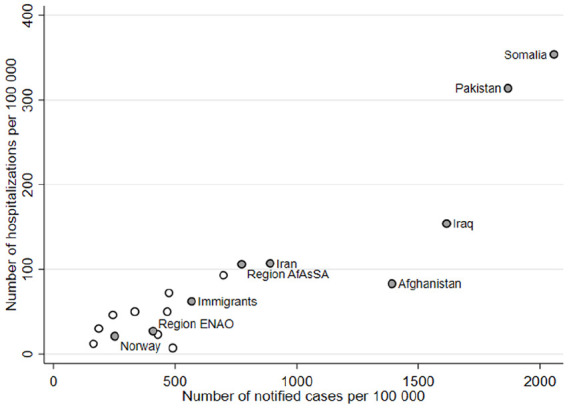
Number of notified infections per 100,000 people versus number of COVID-19-related hospitalizations per 100,000, immigrant groups and non-immigrants.

Hospitalization rates varied markedly with country of birth. Whereas 21 per 100,000 in the non-immigrant population were admitted, the rate was less than a third (seven per 100,000) in the largest immigrant group in Norway – Polish born. In contrast, the hospitalization rate among Somali-born immigrants was 15 times higher than among non-immigrants (354 per 100,000) (Table I). With some exceptions, countries with high numbers of notified cases also had high numbers of hospitalizations (Figure 1).

Syrian- and Thailand-born immigrants were the only groups that had fewer notified cases per 100,000 (243 and 185, respectively) than non-immigrants (251), but higher number of hospitalizations per 100,000 (46 and 30, respectively versus 23) (Table I). Those born in Syria, Iraq, Somalia, Thailand, Denmark, The Philippines, Romania, Iran, Eritrea and Iraq all had higher number of hospitalizations per notified case than non-immigrants (Table I and Supplemental Figure 1). Among immigrants from western Europe (except Denmark), there were relatively few hospitalizations relative to confirmed cases, despite higher ages. Median age at hospitalization was 66 years among non-immigrants and particularly low among immigrants born in Lithuania (38 years), Syria (42 years) and Eritrea (43 years) (Table I).

**Figure 2.**
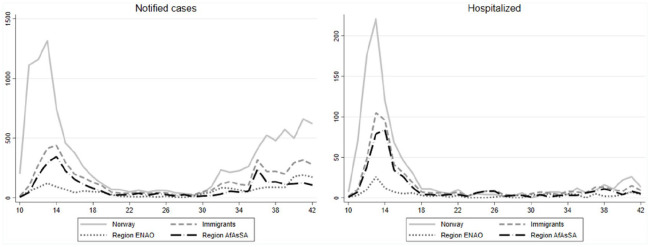
Timeline for numbers of notified cases and COVID-19-related hospitalizations among immigrants and non-immigrants in total and by region.

Immigrants were infected later into the pandemic than non-immigrants, but experienced a comparatively delayed decline (Figure 2). After a decline in both groups in April, the curve for new cases was flattening for both immigrants and non-immigrants from May onwards, before increasing again in all groups after the summer, still with a high proportion of immigrants among notified cases. The same pattern was seen for hospitalizations, but with the proportion of immigrants exceeding non-immigrants some weeks in May and June.

**Figure 3.**
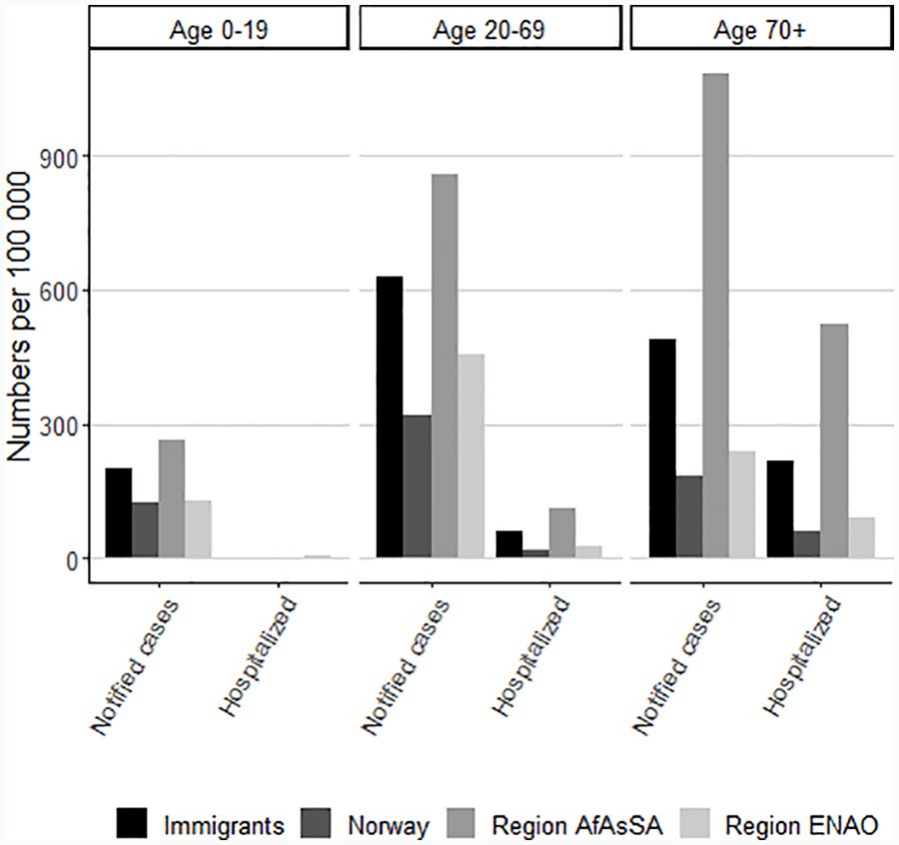
Notified cases and COVID-19-related hospitalizations according to age groups.

**Figure 4.**
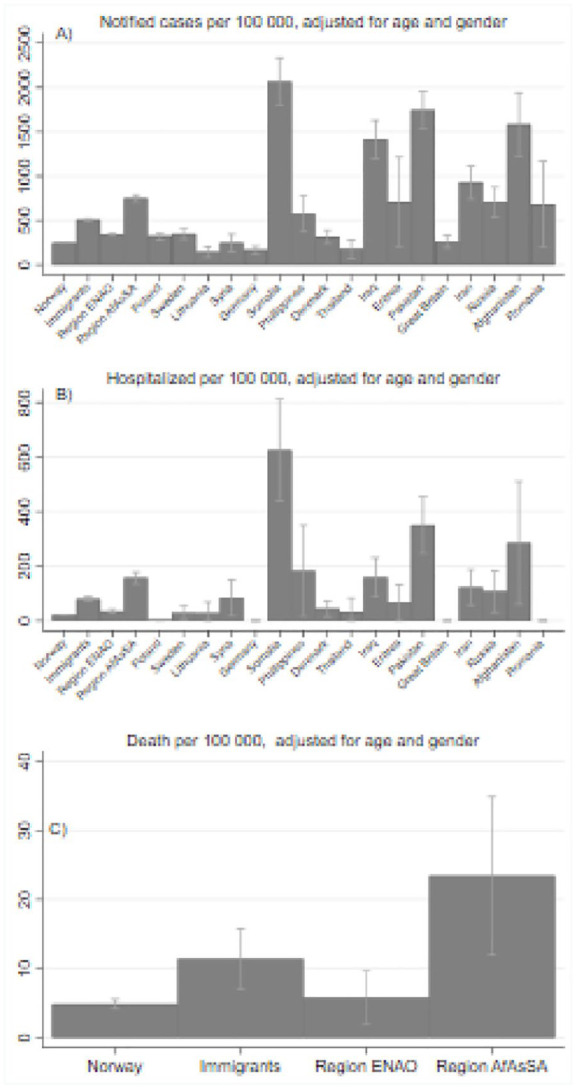
Age- and sex-adjusted numbers (per 100,000) for notified cases, hospitalizations and death for each immigrant group. Numbers <5 not shown.

Rates of notified cases were highest in the 20–69 year age group both among non-immigrants and immigrants from the ENAO region (Figure 3). The rates of notified cases were highest among immigrants from AfAsSA in all age groups, but highest in the oldest age group, probably because of a small total number of people in this age group from this region. The rates of hospitalization were highest among persons aged ⩾70 years among both non-immigrants and immigrants from both regions (Figure 3). After age and sex adjustment, the excess burden of notified cases of COVID-19 among immigrants was not markedly altered in the groups with the highest notification rates (Somalia, Pakistan and Iraq), but slightly increased in groups with low median age, such as Afghanistan and Eritrea. The excess rates of hospitalization increased in many immigrant groups when adjusted for age and sex (Figure 4). We observed no excess toll of COVID-19-associated mortality among immigrants compared to non-immigrants before adjusting for age (four versus five per 100,000). After adjustment for sex and age, this gap increased to 11 among immigrants and 23 among immigrants from AfAsSA versus five per 100.000 among non-immigrants (Figure 4). Mean age at death was 86 years among non-immigrants, 77 among immigrants, 85 years among immigrants from region ENAO and 76 years among immigrants from AfAsSA.

There was no notable difference in rate of notified cases among immigrants compared to non-immigrants in Oslo compared to outside Oslo. The number of confirmed cases per 100,000 people in Oslo was 650 among persons born in Norway, 650 among immigrants from ENAO and 1450 among immigrants from AfAsSA (Supplemental Figure 2). The corresponding numbers outside Oslo were 250, 300 and 550.

## Discussion

Immigrants had higher rates of notified COVID-19 and related hospitalizations than non-immigrants. Due to low total numbers and young age in many immigrant groups, it is not similarly clear whether immigrants should be considered at higher risk for mortality from COVID-19 in Norway. But if we adjust for age, the immigrants from AfAsSA have substantially higher mortality rates than non-immigrants.

There is a clear tendency that immigrant groups with high numbers of notified cases also had high numbers of hospitalizations, and vice versa. Because hospitalizations are unlikely to depend on test criteria, this tendency suggests a strong positive correlation between notified and actual cases. For some immigrant groups, however, there is a high number of hospitalizations per notified case. This might indicate a higher level of unconfirmed cases in these groups, although it may also be related to underlying factors that increase the risk of serious complications in these population groups. Moreover, we also reported a high age-adjusted rate of COVID-19-related mortality among immigrants from Asia, Africa and Latin America, corresponding to groups with high rates of hospitalizations.

The higher rates of notified COVID-19 cases among certain groups that we find in Norway follow some of the same patterns as reported in Sweden [18], where the numbers per 100,000 were high for Somali-, Iraq- and Iran-born immigrants. Also in Denmark are immigrants from non-Western countries, especially Somalia, Pakistan and Turkey, who have a particularly high incidence of COVID-19 [3]. Numbers from Sweden, Denmark and Norway are taken at different date intervals and can therefore not be directly compared. The results also resemble what is reported among ethnic minorities in the United Kingdom, with overrepresentation of infected cases and hospitalizations among Black and Asian residents [5,8].

The above-mentioned differences between groups might have been caused by a mix of factors that in isolation contribute with only minor effects. Yet when such factors are combined, they may cause observable differences due to their common relation to immigration. We did not have data on such factors in this study, but we propose three groups of factors likely to be involved, which should be investigated further in a Norwegian context. Differences among immigrant groups and between immigrants and non-immigrants in notified cases, associated hospitalizations and mortality may originate from, for example, (a) differences in exposure, such as travel or living conditions; (b) underlying factors affecting the severity of and mortality from infection, such as age distribution and medical conditions; and (c) factors influencing the chance of getting diagnosed, such as test criteria and access to health services.

### Differences in exposure

Immigrants are over represented in low educational groups, in low-income and temporary employment positions and in occupations with repeated close contact with the public [19–21]. Such working conditions may force people to work, even when infection-control measures are hard to follow at the workplace or when commuting to work. Some might also be hesitant to test for COVID-19 infection because of fear of losing their position if absent. However, in our study, the groups with few notified cases and hospitalizations consist of people who are typically labour migrants. The highest rates of COVID-19 is found among people of working age and it has been suggested that high rates among immigrants partly can be explained by high proportions of households of working age [1,22]. A study in Sweden linked occupations often held by immigrants to an increased risk of COVID-19 [23]. Further, Norwegian data suggest persons with low education and low income face most barriers to keep official advice regarding social distancing and reduced travelling [24].

Small apartments and/or larger households may make it difficult to practice social distancing, although studies report differently regarding the role of crowded housing in the spreading of the virus [5,25]. A high proportion of immigrants living in crowded housing could be a part of the explanation for high incidence rates of notified cases in some groups. International studies assessing the importance of socioeconomic factors in the spread of COVID-19 among ethnic minorities and immigrants report that such factors contribute but cannot fully explain the differences in incidence rates observed between minority groups or immigrants compared to host population [8,9].

Large cities often have large proportions of immigrants, and urban living could be a factor in the spread of COVID-19 [5]. However, in our study, higher notification and hospitalization rates among immigrants could not be explained by their overrepresentation in Oslo as the epicentre for COVID-19 in Norway. In fact, the high rate of notified COVID-19 among immigrants compared to non-immigrants was almost as strong outside Oslo as in Oslo. Further, differences in international travel patterns may explain some of the differences we observe between groups.

### Underlying factors

People with underlying poor health have an increased risk of severe COVID-19 [26]. Health is not equally distributed among immigrants. Many arrive especially healthy, but with increased duration of stay in the host country, many experience a deterioration of health. Health is also shown to deteriorate at a younger age among immigrants than in the host population [27,28]. In Norway, immigrant groups from South Asia and the Middle East, together with women from Somalia, have shown to have a high prevalence of obesity [28,29], diabetes [28,30] and cardiovascular disease [31,32], all predisposing for severe COVID-19 [24,33]. Social disadvantage, as experienced by many immigrants, has also been linked to increased risk of COVID-19 [5,6]. Previous studies indicate that socioeconomic factors or indicators of physical or mental health cannot fully explain ethnic differences in the burden of COVID-19 [8,9]. Associations between COVID-19 and socioeconomic factors may vary with circumstances and it will be important to investigate these relationships further in future research.

### Factors influencing chance of getting diagnosed

Changes in testing criteria, resources and policy may explain some of the differences in the notification rates we observe between groups, but less so for hospitalization rates because all people developing severe COVID-19 are likely to be hospitalized and diagnosed. In the early phase of the pandemic, Norway observed much stricter testing criteria than later in the pandemic. Initially, only symptomatic persons returning from high incidence countries were offered a test, whereas now anybody with symptoms of infection in the airways, and even some groups without symptoms, are urged to get tested. Immigrants may experience a range of barriers, including language, to test for COVID-19 and to seek and receive adequate healthcare. Cultural and social practices, fear and stigma connected with being diagnosed could also play a role in some groups.

### Strengths and limitations

We have taken advantage of register data, with national coverage and linkage to country of birth, and have information on all notified cases of COVID-19 in Norway and associated hospitalization and mortality. We have no information on country of birth for persons who have tested negative for COVID-19. Thus, we cannot estimate proportions tested in each group or number of positive tests relative to total number of tests. There were differences between groups in number of notified cases relative to COVID-19-related hospitalizations. This could suggest differences between groups in severity of infection, in age distributions, in underlying health and factors related to exposure and in test criteria and behaviour. Differences in test criteria might produce sampling bias, as this might have led to some groups being tested more than others. Our measures of hospitalization and mortality may include persons hospitalized for, or dying from, other reasons than COVID-19. We had no access to sociodemographic variables at the individual level or health-related information and could not assess the importance of such variables in differences between immigrants and non-immigrants in notified cases of COVID-19.

### Implications

Norwegian authorities and municipalities have initiated and financed a number of measures to reach the immigrant population with information and advice on COVID-19. This includes translated information material and information campaigns. Various immigrant organizations and community leaders have promoted and actively worked toward immigrant communities to prevent the spread of infection. A major surge in COVID-19 cases among Somalis in late March and early April 2020 was brought to halt in a short time, possibly due to the combined efforts of national and local government and the Somali-Norwegian community. The effect of these types of efforts need to be investigated in further detail. Our findings suggest the need for more and better measures to prevent infection among immigrant populations. Possible barriers to appropriate information due to low health literacy in certain groups and misconceptions about COVID-19, testing criteria etc. need to be better understood. Further, the role of socioeconomic and environmental factors needs to be assessed. Of special interest is the role of occupation and risk due to exposure at work and other barriers to following social distancing, such as crowded housing, economy and connection to the labour market, need for public transport and language barriers to information and health services. High rates of notified cases and the high number of associated hospitalizations in some groups indicate that hospitals and other health services should plan for and adapt to the needs of immigrant patients.

## Conclusion

After the first weeks of the pandemic, immigrants in Norway have had higher rates than Norwegian-born residents of notified COVID-19 infections and related hospitalization, but with major differences across immigrant groups. Differences between immigrants and Norwegian-born groups increased when adjusting for age, especially for mortality rates. The relationship between notified infections and hospitalizations could indicate a higher level of unconfirmed cases in some groups. The much higher toll of notified COVID-19 infections among several immigrant groups compared to non-immigrants suggests a need for actions such as enhancing community engagement and health communication strategies to lower the thresholds for being tested. These actions should be appropriately evaluated.

## Supplemental Material

sj-pdf-1-sjp-10.1177_1403494820984026 – Supplemental material for COVID-19 among immigrants in Norway, notified infections, related hospitalizations and associated mortality: A register-based study Click here for additional data file. Supplemental material, sj-pdf-1-sjp-10.1177_1403494820984026 for COVID-19 among immigrants in Norway, notified infections, related hospitalizations and associated mortality: A register-based study by Thor Indseth, Mari Grøsland, Trude Arnesen, Katrine Skyrud, Hilde Kløvstad, Veneti Lamprini, Kjetil Telle and Marte Kjøllesdal in Scandinavian Journal of Public Health

## References

[1] Drefahl S Wallace M Mussino E, et al Socio-demographic risk factors of COVID-19 deaths in Sweden: A nationwide register study. Stockholm Research Reports in Demography 2020:23 Stockholm University, 2020.

[2] Hansson E Albin M Rasmussen M, et al Stora skillnader i överdödlighet våren 2020 utifrån födelseland [Large differences in excess mortality spring 2020 according to country of birth] Läkartidningen. 2020;117:20113 32619245

[3] Statens Serum Institut. COVID-19 og herkomst – opdateret fokusrapport [COVID-19 and country of origin. An updated focus report]. Sundhetsministeriet, 2020.

[4] Lauvrak V Juvet L. Social and economic vulnerable groups during the COVID-19 pandemic, Rapid review 2020. Oslo: Norwegian Institute of Public Health, 2020.

[5] de Lusignan S Dorward J Jones CA, et al Risk factors for SARS-CoV-2 among patients in the Oxford Royal College of General Practitioners Research and Surveillance Centre primary care network: A cross-sectional study. The Lancet Infectious Diseases 2020:S1473-3099(20)30371-6. doi: 10.1016/S1473-3099(20)30371-6. Online ahead of print. PMC7228715 32422204

[6] Williamson EJ Walker AJ Bhaskaran K, et al OpenSAFELY: Factors associated with COVID-19-related hospital death in the linked electronic health records of 17 million adult NHS patients. Nature 2020;584:430–436. 32640463 10.1038/s41586-020-2521-4 PMC7611074

[7] Aldridge RW Lewer D Katikireddi SV, et al Black, Asian and Minority Ethnic groups in England are at increased risk of death from COVID-19: Indirect standardisation of NHS mortality data. Wellcome Open Res 2020;24:88. 10.12688/wellcomeopenres.15922.1 PMC7317462 32613083

[8] Lassale C Gaye B Hamer M, et al Ethnic disparities in hospitalisation for COVID-19 in England: The role of socioeconomic factors, mental health, and inflammatory and pro-inflammatory factors in a community-based cohort study. Brain Behavior Immunity 2020;88:44–49. 10.1016/j.bbi.2020.05.074 PMC7263214 32497776

[9] Niedzwiedz CL O’Donnell CA Jani BD, et al Ethnic and socioeconomic differences in SARS-CoV-2 infection: Prospective cohort study using UK Biobank. BMC Medicine 2020;18:16. 32466757 10.1186/s12916-020-01640-8 PMC7255908

[10] El C Maher King K, et al Are African American and Hispanics disproportionately affected by COVID-19 because of higher obesity rates? Surg Obes Relat Dis 2020;16:1096–1099. 32522406 10.1016/j.soard.2020.04.038 PMC7211681

[11] Mollalo A Vahedi B Rivera KM. GIS-based spatial modeling of COVID-19 incidence rate in the continental United States. Sci Total Environ 2020;728:138884. 32335404 10.1016/j.scitotenv.2020.138884 PMC7175907

[12] European Centre for Disease Prevention and Control. 2020 COVID-19 country overviews. https://COVID19-country-overviews.ecdc.europa.eu/ (Accessed 19 October 2020).

[13] FOR-2003-06-20-740. Forskrift om Meldingssystem for smittsomme sykdommer [Regulation of Norwegian Surveillance System for Communicable Disease]. https://lovdata.no/dokument/SF/forskrift/2003-06-20-740?q=MSIS. (Accessed 4 June 2020).

[14] Folkehelseinstituttet. Meldingskriterier for sykdommer i MSIS [Criterias for notification of disease in MSIS]. http://www.fho.no/publ/2017/meldingskriterier-for-sykdommer-i-msis/ (Accessed 1 May 2020).

[15] Folkehelseinstituttet. MSIS meldingsskjema. Nominativ melding om smittsom sykdom. [notification to MSIS, nominative notification of communicable disease] https://www.fhi.no/publ/2019/msis-meldingsskjema.-nominativ-meld/ (Accessed 1 May 2020).

[16] Folkehelseinstituttet. Beredskapsregisteret for COVID-19 [Preparednessregister for COVID-19] https://www.fhi.no/sv/smittsomme-sykdommer/corona/norsk-beredskapsregister-for-COVID-19/ (Accessed 28 August 2020).

[17] Brurberg K Fretheim A. COVID-19: The relationship between age, comorbidity and disease severity – a rapid review. [COVID-19: Sammenheng mellom alder, komorbiditet og sykdomsalvorlighet – en hurtigoversikt. Hurtigoversikt 2020.] Oslo: Norwegian Institute of Public Health, 2020

[18] Folkhälsomyndigheten. COVID-19. Demografisk beskrivning av bekräftade COVID-19 fall i Sverige 13 mars-7 maj 2020. [Demographic description of confirmed cases of COVID-19 in Sweden 13 March to 7 May] Sweden, Folkhälsomyndigheten 2020. Artikelnummer 20096.

[19] Statistics Norway. SSB Analyse 2020/14 Midlertidige ansettelser [Analysis 2020/14. Temporary positions]. https://www.ssb.no/arbeid-og-lonn/artikler-og-publikasjoner/flere-innvandrere-jobber-i-en-midlertidig-stilling. (Accessed 19 October 2020).

[20] Hawkins D. Differential occupational risk for COVID-19 and other infection exposure according to race and ethnicity. Am J Ind Med 2020;63:817–820. 32539166 10.1002/ajim.23145 PMC7323065

[21] Office for National Statistics. Which occupations have the highest potential exposure to the coronavirus (COVID-19)? https://www.ons.gov.uk/employmentandlabourmarket/peopleinwork/employmentandemployeetypes/articles/whichoccupationshavethehighestpotentialexposuretothecoronavirusCOVID19/2020-05-11. (Accessed 19 October 2020).

[22] Rostila M Cederström A Wallace M, et al Disparities in COVID-19 deaths by country of birth in Stockholm, Sweden: A total population based cohort study. Stockholm Research Reports in Demography 2020 Preprint. 10.17045/sthlmuni.12852854.v1 PMC7989658 33710317

[23] Folkehälsomyndigheten. Förekomst av COVID-19 i olika yrkesgrupper. Bekräftade COVID-19 fall i Sverige 13 mars - 27 maj 2020. [Prevalence of COVID-19 in different occupational groups. Confirmed cases in Sweden 13 March to 27 May] Folkhälsomyndigheten 2020. Artikelnummer 20099.

[24] Ingelsrud M, H Ellingsen D Steen AH. Arbeidslivsbarometer. Norsk arbeidsliv 2020. Hele Norge på dugnad – konsekvenser og konstanter. [Norwegian worklife 2020] https://s32603.pcdn.co/wp-content/uploads/2020/08/Arbeidslivsbarometeret2020_Hele-Norge-paa-dugnad.pdf.

[25] Raisi-Estabragh Z McCracken C Bethell MS, et al Greater risk of severe COVID-19 in Black, Asian and Minority Ethnic populations is not explained by cardiometabolic, socioeconomic or behavioural factors, or by 25(OH)-vitamin D status: Study of 1326 cases from the UK Biobank. J Public Health 2020;42:451–460. 10.1093/pubmed/fdaa095 PMC7449237 32556213

[26] Yang J Zheng Y Gou X, et al Prevalence of comorbidity and its effect in patients infected with SARS-CoV-2: A systematic review and meta-analysis. Int J Infect Dis 2020;94:9–95. 10.1016/j.ijid.2020.03.017 PMC7194638 32173574

[27] World Health Organization. Report on the health of refugees and migrants in the WHO European Region. No public health without refugee and migrant health. Copenhagen: World Health Organization; 2018.

[28] Kjøllesdal M Straiton ML Øien-Ødegaard C, et al Helse blant innvandrere i Norge. Levekårsundersøkelsen blant innvandrere 2016 [Health among immigrants in Norway. The living condition survey among immigrants 2016]. Oslo: Norwegian Institute of Public Health 2019.

[29] Ahmed SH Meyer HE Kjøllesdal MK, et al Prevalence and predictors of overweight and obesity among Somalis in Norway and Somaliland. A comparative study. J Obes 2018. doi: 10.1155/2018/4539171. PMC6140005 30250753

[30] Jenum AK Diep LM Holmboe-Ottesen G, et al Diabetes susceptibility in ethnic minority women groups from Turkey, Vietnam, Sri Lanka and Pakistan compared with Norwegians: The association with adiposity is strongest for ethnic minority women. BMC Public Health 2012;12:1–12. 22380873 10.1186/1471-2458-12-150 PMC3315409

[31] Rabanal KS Lindman AS Selmer RM, et al Ethnic differences in risk factors and total risk of cardiovascular disease based on the Norwegian CONOR study. Eur J Prev Cardiol 2013;20:1013–1021. 22642981 10.1177/2047487312450539

[32] Rabanal KS Selmer R Igland J, et al Ethnic inequalities in acute myocardial infarction and stroke rates in Norway 1994–2009: A nationwide cohort study (CVDNOR). BMC Public Health. 2015;20:1073. 10.1186/s12889-015-2412-z PMC4612407 26487492

[33] Zheng Z Peng F Xu B, et al Risk factors of critical and mortal COVID-19 cases: A systematic literature review and meta-analysis. J Infect 2020:81;e16–e25. 10.1016/j.jinf.2020.04.021 PMC7177098 32335169

