# Supplementary material for: COVID-19 among immigrants in Norway, notified infections, related hospitalizations and associated mortality: A register-based study
Source: Scand J Public Health. 2021 Jan 7;49(1):48–56. doi: 10.1177/1403494820984026 (PMC7859570; doi:10.1177/1403494820984026)

Supplemental figure 1. Hospitalizations per 100 notified cases in each immigrant group, countries with less than 5 cases are not included.

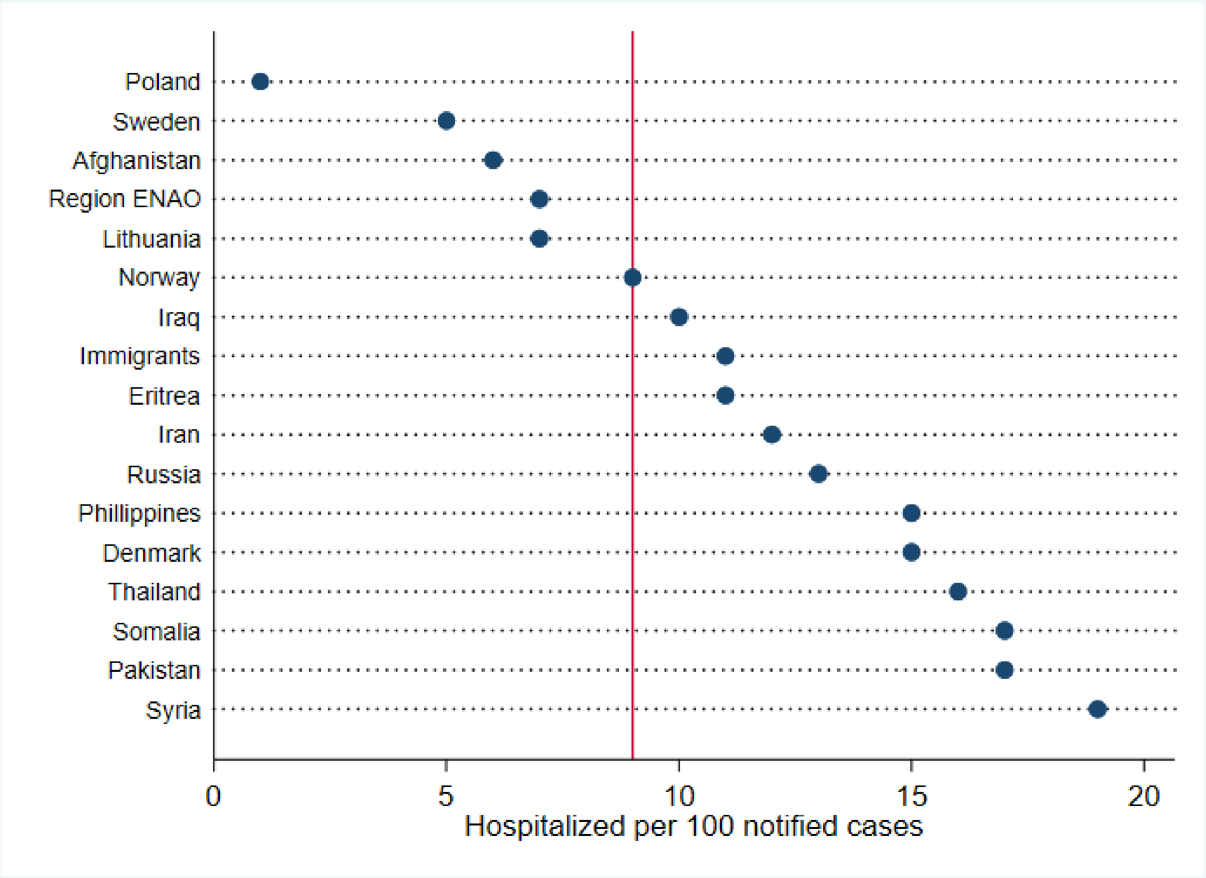

Supplemental figure 2. Notified cases per 100.000 among non-immigrants and immigrants in total and by region in and outside Oslo.

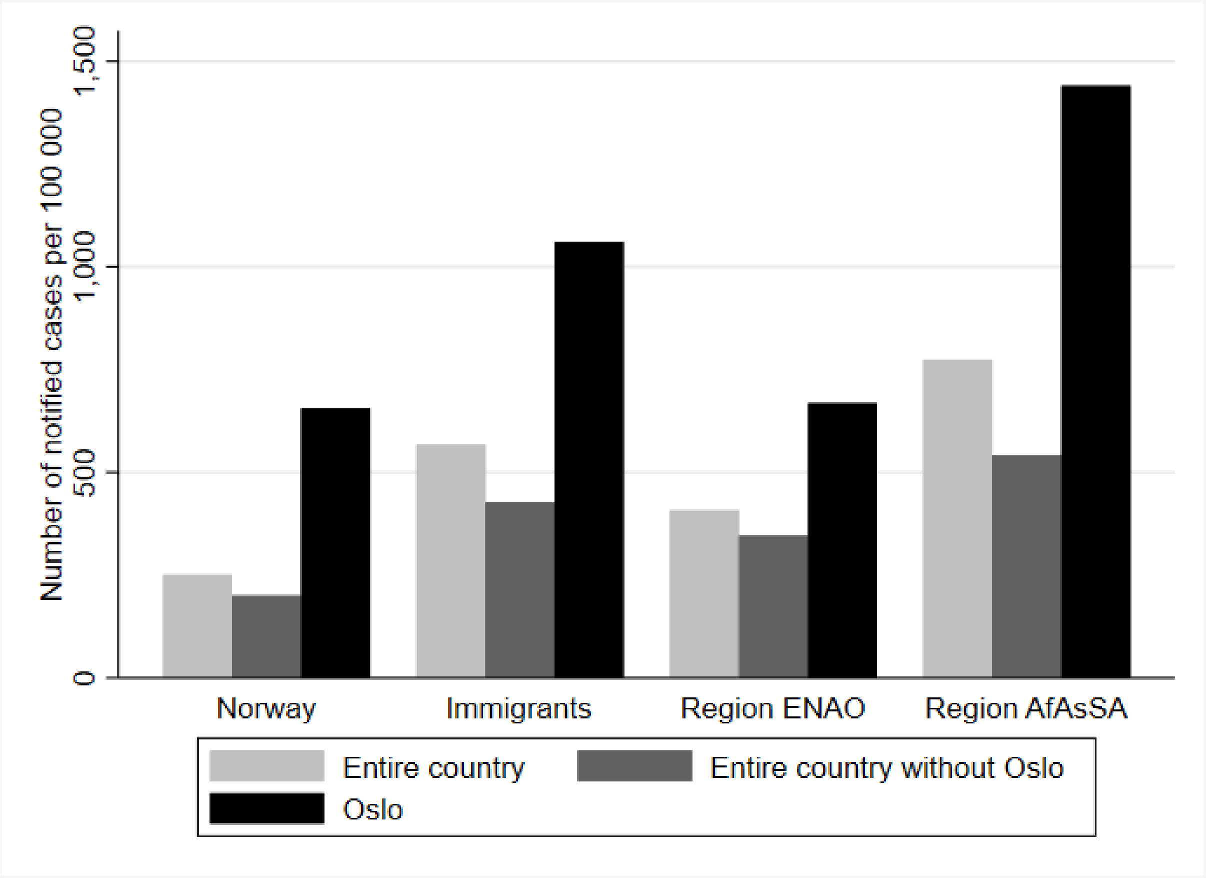

Supplement: sj-pdf-1-sjp-10.1177_1403494820984026 – Supplemental material for COVID-19 among immigrants in Norway, notified infections, related hospitalizations and associated mortality: A register-based study [file sj-pdf-1-sjp-10.1177_1403494820984026.pdf]
